# Protective effects of gallic acid against nickel-induced kidney injury: impact of antioxidants and transcription factor on the incidence of nephrotoxicity

**DOI:** 10.1080/0886022X.2024.2344656

**Published:** 2024-04-29

**Authors:** Areej I. Alhazmi, Mohamed F. El-Refaei, Eman A. A. Abdallah

**Affiliations:** a Faculty of Medicine, Al-Baha University, Al Baha, Saudi Arabia; b Biochemistry and Molecular Biology, Genetic Institute, Sadat City University, Sadat City, Egypt; c Forensic Medicine and Clinical Toxicology, Faculty of Medicine, Zagazig University, Egypt

**Keywords:** Gallic acid, antioxidant, transcription factor, nickel, nephrotoxicity

## Abstract

Nickel (Ni) is a common metal with a nephrotoxic effect, damaging the kidneys. This study investigated the mechanism by which gallic acid (GA) protects mice kidneys against renal damage induced by Nickel oxide nanoparticles (NiO-NPs). Forty male Swiss albino mice were randomly assigned into four groups, each consisting of ten mice (*n* = 10/group): Group I the control group, received no treatment; Group II, the GA group, was administrated GA at a dosage of 110 mg/kg/day body weight; Group III, the NiO-NPs group, received injection of NiO-NPs at a concentration of 20 mg/kg body weight for 10 consecutive days; Group IV, the GA + NiO-NPs group, underwent treatment with both GA and NiO-NPs. The results showed a significant increase in serum biochemical markers and a reduction in antioxidant activities. Moreover, levels of 8-hydroxy-2’-deoxyguanosine (8-OH-dG), phosphorylated nuclear factor kappa B (p65), and protein carbonyl (PC) were significantly elevated in group III compared with group I. Furthermore, the western blot analysis revealed significant high NF-κB p65 expression, immunohistochemistry of the NF-κB and caspase-1 expression levels were significantly increased in group III compared to group I. Additionally, the histopathological inspection of the kidney in group III exhibited a substantial increase in extensive necrosis features compared with group I. In contrast, the concomitant coadministration of GA and NiO-NPs in group IV showed significant biochemical, antioxidant activities, immunohistochemical and histopathological improvements compared with group III. Gallic acid has a protective role against kidney dysfunction and renal damage in Ni-nanoparticle toxicity.

## 1. Introduction

Acute kidney injury (AKI) is a medical condition that can be described by a reversible decrease in the glomerular filtration rate over hours to days, affecting approximately 13.3 million people annually in underdeveloped nations [1]. Acute kidney injury is associated with higher mortality and morbidity rates, as well as the development of chronic renal disease. Treating the underlying issue reduces these risks. A primary cause of AKI, resulting from nephron injury that is either direct or secondary to ischemia, is exposure to toxins [2]. Toxins vary in mode of action, histopathology, and specific immunologic markers, which facilitates treatment and aids in identifying the therapeutic target [3].

Nanotechnology is one of the fastest -growing areas in science. Particles sized at 100 nm or less are known as nanoparticles (NPs) [4]. Public concern about the biological and medical impacts of NPs has increased due to their growing use in commercial and biomedical applications. Nanoparticles enter the plasma membrane during endocytosis through several direct permeation pathways, dependent on their physicochemical characteristics [5].

Reactive oxygen species (ROS), indicators of oxidative stress, have been identified as a crucial mechanism for the cytotoxicity of nanomaterials, and this has been proposed as the primary mechanism of nickel (Ni) nanoparticle-induced cell damage. Cellular deterioration, inflammation, cell cycle arrest, chromosomal changes, and apoptosis have all been linked to nickel nanoparticles (Ni-NPs) [6,7].

Recently, nickel oxide nanoparticles (NiO-NPs) have been used in a wide range of advanced industrial applications, including nanofibers, electrochromic devices, lithium-ion microbatteries, and various biological technologies. Nickel oxide nanoparticles are categorized as highly dangerous compounds. Frequent and persistent exposure can have severe impacts on human and animal health [8]. The minimal risk levels (MRLs) for nickel exposure that induces chronic lung inflammation, fibrosis, and alveolar proteinosis is 1 x 10^5^ mg Ni/m^3^. However, the oral dose considered lethal is 2.2 mg/kg/day [9].

The liver, kidneys, brain, lungs, and testicles are the organs most frequently exposed to Ni [10]. Nickel oxide nanoparticles are thought to cause cytotoxicity primarily by increasing ROS levels [11], in addition to stimulating cell degeneration and inflammation [12]. Nickel oxide nanoparticles have also been found to cause cytogenetic changes and apoptosis [13]. Moreover, they have been discovered to cause kidney damage by reducing both enzymatic and nonenzymatic antioxidant activities [14] and by suppressing the immune system or activating various inflammatory mediators [15]. Fischer et al. conducted a study on the potential health risks associated with long-term environmental exposure to nickel. According to their research, Ni is linked to a decline in renal function. Greater fold-increase in blood creatinine over baseline (*p* = 0.002) and lower eGFRs (*p* = 0.001) were correlated with higher Ni concentrations [16].

Nuclear factor-kappa B (NF-κB) expression can be stimulated by the direct activation of proinflammatory intracellular signal transduction cascades by Ni^2+^ [17]. Nuclear factor-kappa B (NF-κB) is a family of inducible transcription factors that regulates many genes involved in different pathways of the immune and inflammatory responses [18]. This family involves five structurally related members: NF-B1 (also known as p50), NF-B2 (p52), RelA (p65), RelB, and c-Rel. These members interact with the B enhancer, a specific DNA element, in either heterodimeric or homodimeric configurations to regulate the transcription of target genes [19–21].

Antioxidants from nature are an excellent source for promoting health, as they prevent many unfavorable changes that may otherwise lead to cell deterioration and, consequently, potential disease symptoms. Among these antioxidants, gallic acid (GA) has long been used as a therapeutic agent. Gallic acid (3,4,5-trihydroxybenzoic acid) is a polyphenol that is commonly found in foods such as gallnuts, apple peels, oak bark, tea leaves, grapes, sumac, mango, lemon, and various berries. It is also present in areca nuts, pineapples, strawberries and bananas [22]. Due to its significant antioxidant properties, numerous disorders can be prevented or treated using GA and its derivatives, as they have demonstrated a wide spectrum of beneficial effects [23]. In an acute oral toxicity study of GA, no signs of lethal toxicity were observed at dose up to 5000 mg/kg p.o. In a subacute toxicity study, GA was also deemed safe at 1000 mg/kg p.o. [24].

The current study aims to assess the GA ability to protect against and prevent acute toxicity caused by NiO-NPs *in vivo*. This will be achieved by estimating biochemical markers and antioxidant activities in mouse serum and kidney homogenate. Moreover, Western blot analysis of transcription factor NF-κB p65 will be applied to estimate and examine p65 expression and its role in the redemption of kidney cell injury *in vivo*. Also, biomarker of oxidative DNA damage (8-OH-dG) and protein carbonyl (PC) will be assessed. Additionally, the extent of injury will be studied by determining caspase-1, which revealing the actuality role as a key marker for apoptosis. Furthermore, the histopathology of the kidneys in each group of mice will be considered to elucidate GA’s mode of action and its protective properties in detail.

## 2. Materials and methods

### 2.1. Animals

All procedures were followed in compliance with the relevant regulations and rules. Every technique is reported in accordance with ARRIVE guidelines.

Forty adult male Swiss albino mice (26 ± 2 g) (8–9 weeks age) from the Animal House at King Abdulaziz University in Jeddah, Saudi Arabia, were utilized in the experiment. The mice were housed and adapted for 7 days at the animal house of the Biochemistry Department of the Faculty of Medicine, Al-Baha University. The mice were given unrestricted access to food and drink while they were housed in a room-temperature setting with moderate humidity (60% ± 5%) and 12-h light/dark cycles. Water and oriental chow pellet food were available to the mice *ad libitum*. Male mice were used in this study to eliminate sex-related differences. All experimental protocols were approved by, Faculty of Medicine, Al-Baha University Ethical Committee (Approval number: REC/MED/BU-FM/2023/09).

### 2.2. Chemicals

#### 2.2.1. Nickel oxide nanoparticles (NiO-NPs)

Nickel oxide nanoparticles were bought from Sigma-Aldrich Co. (St. Louis, MO). Sterilized normal saline was used to dissolve them. A toxic dose of (20 mg/kg body weight [b.wt]) NiO-NPs [25] was calculated and injected in the mice *via* the intraperitoneal (i.p.) route for 10 successive days. Kidney function tests and histological examinations were used to validate the induction of nephrotoxicity in the mice groups.

#### 2.2.2. Gallic acid

GA was purchased from Sigma-Aldrich Co. (149-91-7), set with different serial concentrations and doses, the LD_50_ establish at 1800 mg/kg/day. The effective dose was 110 mg/kg/day b.wt (dissolved in distilled water, 0.3 mL), injected for 14 consecutive days [26].

### 2.3. Experimental design

The animals were classified into four groups (*n* = 10 for each group): Group I (negative control group) included 10 mice that were given tap water and a standard diet in order to measure the basic parameters. Group II included 10 mice that received a single daily intraperitoneal injection of GA at a dose of 110 mg/kg/b.wt. Group III (NiO-NPs) included 10 mice, and each mouse received a single daily intraperitoneal injection of NiO-NPs at a dose of 20 mg/kg/b.wt for 10 consecutive days. Group IV (NiO-NPs plus GA) included 10 mice that received NiO-NPs and GA daily, with NiO-NPs intoxication at 20 mg/kg/b.wt. for 10 days and GA at a dose of 110 mg/kg/b.wt. starting from day 1 up to day 14. For biochemical studies, venous blood samples were taken from the retro-orbital plexus of mice using microcapillary glass tubes. At 24 h after the last treatment, all animals were put to death with a sodium thiopental injection intraperitoneally (25 mg/kg) while completely sedated [27]. Kidney tissue samples were then taken for determining oxidative stress activities, immunohistochemical and histopathological studies.

### 2.4. Determination of blood urea nitrogen and creatinine levels in the serum mice groups

Spectrophotometric measurements of blood urea nitrogen (BUN) and serum creatinine levels were performed using Urea Assay Kit (ab83362) and Creatinine Assay Kit (BA0034). Fawcett and Scott’s method [28] was used to assess the serum urea, and the serum creatinine concentration was also quantified in accordance with the method of Henry et al. [29].

### 2.5. Determination of superoxide dismutase, glutathione-S-transferase, glutathione peroxidase, and malondialdehyde activities in kidney homogenates

The kidney samples from each mouse were removed, washed in ice-cold saline, and then homogenized in 20 mm phosphate buffer saline (pH 7.4). The homogenates were then centrifuged at 3000 rpm for 10 min, and the supernatants were kept at −80 °C. The superoxide dismutase (SOD), glutathione-S-transferase (GST), and glutathione peroxidase (GSH-Px) activities in the homogenates were evaluated [30].

The thiobarbituric acid method was used to spectrophotometrically assess the malondialdehyde (MDA) content in the homogenates. Briefly, 150 μl of supernatant was added to 1 mL of thiobarbituric acid (0.66%) and 1 mL of trichloroacetic acid (17.5%). The solution was well mixed by vortex before it was placed in a hot water bath for 15 min. Then, 1 mL of 70% trichloroacetic acid was added after the solution had cooled for 20 min. Centrifugation was then performed for 15 min at 2000 rpm to obtain the supernatant, which was then utilized to estimate the MDA content [31].

### 2.6. Determination of 8-hydroxy-2’-deoxyguanosine (8-OH-dG), phosphorylated NF-κB and protein carbonyl level (PC) in kidney homogenate

#### 2.6.1. Determination of 8-hydroxy-2’-deoxyguanosine (8-OH-dG) level in kidney homogenates

The 8-hydroxy-2′-deoxyguanosine (8-OH-dG) ELISA Kit (ab285254, K4160) (Waltham, Boston, USA) was utilized, according to Abdel-Wahab and Metwally. The test is ­competitive and can be applied to the measurement of 8-OH-dG in tissue homogenate. Both free and DNA-incorporated 8-OH-dG are recognized by it. The assay depends on the competition between 8-OH-dG and 8-OH-dG-acetylcholinesterase (ACHE) conjugate (8-OH-dG Tracer) for a limited amount of 8-OH-dG monoclonal antibody [32].

#### 2.6.2. Measurement of phosphorylated NF-κB in kidney homogenates

Using ELISA kits, the amounts of phospho-NF-κB in the kidney homogenate were determined in accordance with the manufacturer’s instructions. The source of phospho-NF-κB ELISA Kit (ab176648) (Waltham, Boston, USA) [33].

#### 2.6.3. Protein carbonyl assay in kidney homogenates

The protein carbonyl test is based on 2,4 dinitrophenylhydrasine (DNPH) assessment of carbonyl groups’ reactivity. 800 μl of DNPH was combined with two hundred microliters of each protein extract. To precipitate the proteins, we added 1 mL of trichloroacetic acid (TCA 20%) and incubated the tubes for 1 h in the dark, at room temperature. The tubes were then placed in the ice for 10 min and centrifuged for 5 min at 4000 rpm. At 340 nm, a measurement of the optical density was made [34].

### 2.7. Tissue extract preparation and Western blot analysis of NF-κB p65

Kidney samples were homogenized in a T-PER reagent on ice (with sonication for tissues; tissue protein extraction reagent, Pierce Biotechnology, Rockford, IL). As recommended by the manufacturer, the Bio-Rad protein assay reagent (Bio-Rad, Hercules, CA) was employed to determine the entire cell extracts’ protein concentration [35].

Equal amounts of protein samples from the tissue extracts were placed into each well of 10% polyacrylamide-sodium dodecyl sulfate (SDS) gel, and the proteins were then purified using a morpholinepropanesulfonic acid-SDS buffer. After 2 h at room temperature, the proteins were transported to a polyvinylidene fluoride (PVDF) membrane (Millipore-Immobilon-P, Bedford, MA). The nonspecific sites were blocked with 5% dried milk in Tris-buffered saline containing 0.05% Tween-20 after gentle washing for two hours at room temperature (TBST buffer). Membranes were treated with primary NF-κB p65 antibody from Santa Cruz Biotechnology (Santa Cruz, CA) overnight at 4 °C. The primary antibody (NF-kB p65: 1:500 dilution) was diluted in 5% (*w*/*v*) nonfat dry milk in TBST. The PVDF membranes were properly cleaned before incubation for 1 h at room temperature with a secondary immunoglobulin G antibody that had been conjugated with horseradish peroxidase (Santa Cruz Biotechnology, 1:2000 dilution). Using an enhanced chemiluminescence substrate kit, labeled proteins were identified in accordance with the manufacturer’s instructions (Amersham Pharmacia Biotech, GE Healthcare UK Ltd., Bucks, UK) [36].

### 2.8. Immunohistochemistry of NF-κB and caspase-1

#### 2.8.1. Immunohistochemistry of NF-κB

The standard avidin-biotin peroxidase complex method of immunohistochemical staining was used (Vectastain Lab., Inc., Burlingame, CA). Each 4-μm kidney section was first deparaffinized and then rehydrated and incubated for 30 min at room temperature with fresh 0.3% H_2_O_2_ in methanol. The sections were rehydrated using a graded ethanol series and then microwaved in zinc sulfate for 10 min at 90 °C with anti-NF-κB Mab. The sections were then cooled to 30 °C. The sections were first incubated with normal horse serum for 30 min, followed by Mabs at their optimal dilution at 4 °C overnight, followed by phosphate buffered saline washing and secondary antibody incubation at room temperature for 30 min [37].

#### 2.8.2. Immunohistochemistry of caspase-1

In general, caspase-1 was immunohistochemically detected using the streptavidin-biotin immunoperoxidase method. Sections of the kidney were cut to a thickness of 3–5 µm., paraffin was removed with xylene, and the tissue sections were rehydrated with graded alcohol on positively charged slides. After 20 min of heating in buffered citrate (pH 6.0), the sections were then washed in PBS (pH 7.3). By using 6% H_2_O_2_ in methanol, endogenous peroxidase activity was suppressed. For immunohistochemical staining for caspase-1, there was a rabbit polyclonal antibody available for use [38].

### 2.9. Histopathological examination of the kidneys

The kidney tissue sections were dehydrated in ethanol, cleared in xylol, fixed in a buffered formaldehyde solution (10%) for 24 h, infiltrated with paraffin wax for another hour, and then embedded in paraffin wax. The 5 μm thick paraffin slices were stained with hematoxylin and eosin (H&E). A microscope (Eclipse 80i, Nikon, Japan) was employed to assess the morphological alterations, and video cameras (DS-Fi1 digital microscope camera, Nikon) were utilized to capture the pictures.

### 2.10. Computer-assisted digital image analysis (digital morphometric study)

Slides were photographed with a 20 X objective using the Future WinJoe program and the MVV5000CL digital eyepiece that was set up on a MEIJI MX5200L microscope. The generated 20X pictures were examined using Fiji ImageJ (version 1.51r; NIH, Maryland, USA) software and the color deconvolution 2 plugin (histological dyes digital separation) on an Intel® core I7® based computer to calculate the percentage of the surface area stained by immunohistochemical staining. Three separate digital images were obtained as a result (H&E, DAB, and a complimentary image). For every slide, five randomly selected fields measuring 200 × 200 µm were examined. The extent of histopathological lesions in kidney sections was measured using the EGTI scoring system, which was created particularly for research on injured kidney tissues in animals. The four distinct components of histological injury in this system are tubular, interstitial, glomerular, and endothelial [39].

## 3. Statistical analysis

The data collected for each group were expressed as mean ± standard deviation (SD), and comparisons between the examined groups (more than two groups) were made using one-way analysis of variance. The Tukey test was also used to determine significant group differences. The calculations were made using an IBM computer and the SPSS version 20 software package. *p* values < 0.05 were considered statistically significant, and *p* values < 0.001 were considered highly significant.

## 4. Results

### 4.1. NiO-NPs intoxication and effects of GA on BUN and creatinine serum levels of the mice different groups

**Figure 1.**
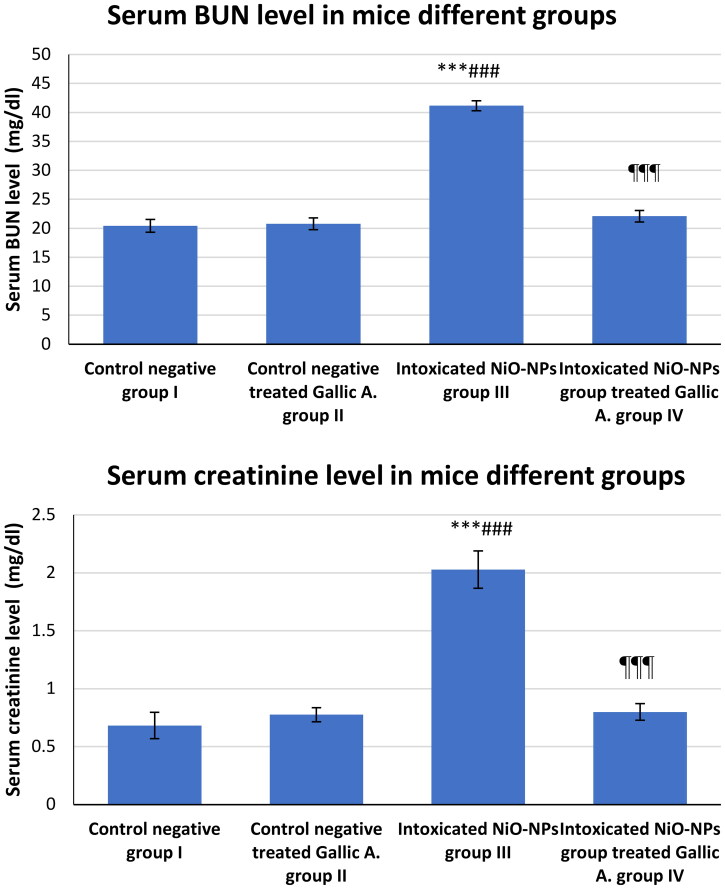
Serum BUN and Creatinine level in mice different groups. *p < 0.05; **p < 0.01; ***p < 0.001 vs. Control negative group I. #*p* < 0.05; ##*p* < 0.01; ###*p* < 0.001 vs. Control negative treated Gallic A. group II ¶*p* < 0.05; ¶¶*p* < 0.01; ¶¶¶*p* < 0.001 vs. Intoxicated NiO-NPs group III.

The BUN and creatinine levels in the serum samples withdrawn from each mouse in all experiment groups were evaluated. In group III, NiO-NPs administration resulted in a significant increase in blood BUN level of 41.16 mg/dl compared to group I (20.41 mg/dl). BUN in group IV showed significant improvement (21.49 mg/dl) as a result of GA treatment compared to group III intoxicated with NiO-NPs. In terms of creatinine levels, the samples from group III showed obvious intoxication, as demonstrated by the increased serum levels of creatinine up to 2.028 mg/dl higher than those in group I, which is 0.682 mg/dl. The results in group IV showed a significant reduction in creatinine level up to 0.799 mg/dl (*p* < 0.001; Figure 1).

### 4.2. NiO-NPs intoxication and effects of GA on SOD, GST, GSH-Px, and MDA activities in the kidney homogenates of the mice different groups

The toxicity of NiO-NPs was evaluated in the current study taking into account the following parameters. Decreased SOD (U/g), GST (U/g), and GSH-Px (U/g) levels and increased MDA (µM/g) were found in group III compared to group I. Significant changes were observed after NiO-NPs inoculation at a dose of 20 mg/kg/b.wt. The SOD level was significantly reduced in group III (32.05 ± 1.74 U/g) compared to group I (63.51 ± 2.09 U/g; *p* < 0.001). The GST and GSH-Px levels were significantly lower in group III (32.02 ± 3.31 U/g and 33.1 ± 1.37 U/g, respectively) than in the control group I (71.76 ± 2.07 U/g and 71.64 ± 1.87 U/g, respectively) (*p* < 0.001). The MDA level in group III (14.8 ± 0.44 µM/g) was significantly higher than that in the control group (8.516 ± 0.376 µM/g) (Table 1).

**Table 1.** SOD level (U/g), GST level (U/g), GSH-Px level (U/g), MDA (µM/g) in mice different groups.

Group/ Parameter	Control negative group I	Control negative treated Gallic A. group II	Intoxicated NiO-NPs group III	Intoxicated NiO-NPs group treated Gallic A. group IV	*p* Value
SOD (U/g)	63.51 ± 2.09	63.19 ±.1.41	32.05 ± 1.74**^*###^	60.96 ± 3.36^¶¶¶^	<0.0001
GST (U/g)	71.76 ± 2.07	70.59 ± 2.69	32.02 ± 3.31^***###^	68.82 ± 1.2^¶¶¶^	<0.0001
GSH-px (U/g)	71.64 ± 1.87	70.25 ± 1.42	33.1 ± 1.37^***###^	70.13 ± 1.41^¶¶¶^	<0.0001
MDA (µM/g)	8.516 ± 0.376	8.622 ± 0.41	14.8 ± 0.44^***###^	8.99 ± 0.88^¶¶¶^	<0.0001

Data expressed as mean ± SD.

SD: standard deviation; P: probability;Test used: One way ANOVA followed by posthoc tukey.

* *p* < 0.05; **p < 0.01; ***p < 0.001 vs. Control negative group I.

^#^ *p* < 0.05; ^##^*p* < 0.01; ^###^*p* < 0.001 vs. Control negative treated Gallic A. group II.

^¶^ *p* < 0.05; ^¶¶^*p* < 0.01; ^¶¶¶^*p* < 0.001 vs. Intoxicated NiO-NPs group III.

The findings showed that GA treatment at a dose of 110 mg/kg/b.wt i.p. in group IV elicited significant increases in SOD, GST, and GSH-Px activity levels, especially when compared to group III (*p* < 0.001). A significant decrease in MDA activity levels was also observed in the GA-treated group IV compared to the NiO-NPs- intoxicated group III (*p* < 0.001; Table 1). On the basis of these findings, GA treatment at a dose of 110 mg/kg/b.wt enhanced the ROS elimination in the mice kidneys. Furthermore, the GA-treated mice group II exhibited no symptoms of agitation, weight loss, sores, or death during the experimental period and investigations.

### 4.3. GA effect on 8-OH-dG, phosphorylated-NF-κB and PC levels in the kidney homogenates of the mice different groups

One often used indicator of oxidative DNA damage is the 8-OH-dG, an oxidative DNA lesion created by oxidation of the C-8 site of 20 deoxyguanosine. The 8-OH-dG in the NiO-NPs group III, were increased compared to control group I. Interestingly, GA- treated group IV reduced 8-OH-dG level significantly compared to intoxicated group III. Also, the levels of phospho-NF-κB in the NiO-NPs group III were significantly increases compared to the control group I (*p* < 0.0001). However, the treated group IV was completely abrogated by GA treatment (*p* < 0.0001). Moreover, PC was considered while assessing the toxicity of NiO-NPs. The data demonstrates an increase in PC in group III compared to group I. On the other hand, a significant decrease in PC level observed in group IV (*p* < 0.0001) (Table 2). No significant changes have been observed between group I and II in these specified parameters throughout the experiments.

**Table 2.** Renal tissue homogenate 8-OH-dG (ng/mg/protein), phospho-NF-κB (pg/ml), and PC (nmol/mg) levels in mice different groups.

Group/ Parameter	Control negative group I	Control negative treated Gallic A. group II	Intoxicated NiO-NPs group III	Intoxicated NiO-NPs group treated Gallic A. group IV	*p* Value
8-OH-dG (ng/mg/protein)	38.478 ± 0.629	39.577 ± 0.523	75.498 ± 1.670^***¶¶¶^	39.850 ± 0.817^*###^	<0.0001
Phospho-NF-κB (pg/ml)	12.026 ± 0.321	12.376 ± 0.614	66.054 ± 5.140^***¶¶¶^	25.753 ± 0.757^***###¶¶¶^	<0.0001
PC (nmol/mg)	0.032 ± 0.003	0.034 ± 0.004	0.485 ± 0.029^***¶¶¶^	0.048 ± 0.005^###^	<0.0001

Data expressed as mean ± SD.

SD: standard deviation; P:probability; Test used: one way ANOVA followed by post-hoc Tukey.

* *p* < 0.05; **p < 0.01; ***p < 0.001 vs. Control negative group I.

^¶^ *p* < 0.05; ^¶¶^*p* < 0.01; ^¶¶¶^*p* < 0.001 vs. Control negative treated Gallic A. group II.

^#^ *p* < 0.05; ^##^*p* < 0.01; ^###^*p* < 0.001 vs. Intoxicated NiO-NPs group III.

### 4.4. Western blot analysis of transcription factor NF-κB p65 expression

**Figure 2.**
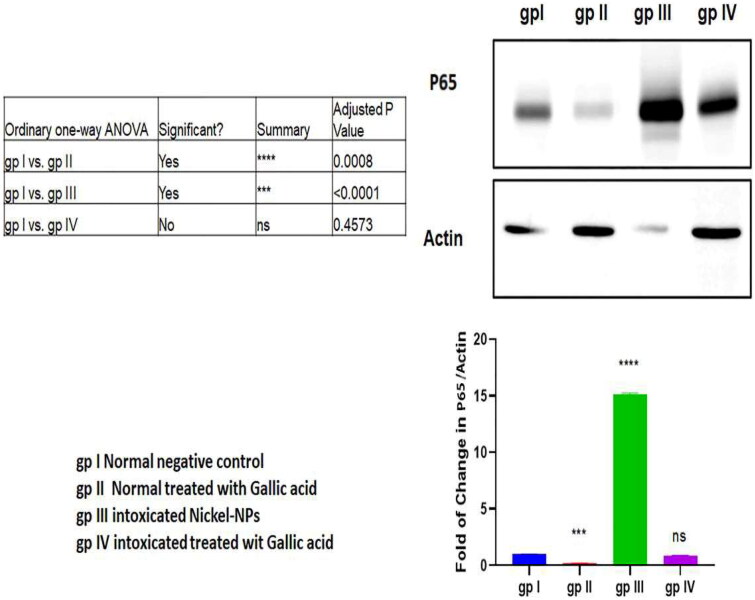
Western blot assay of p65 protein levels in mice different groups.

The western blot analysis revealed significant high NF-κB p65 expression levels in group III than group I (*p* < 0.001). GA administration decreased the NF-κB p65 expression level in group IV compared to group III (*p* < 0.001; Figure 2).

### 4.5. Immunohistochemical results (light microscopic detection of NF-κB and caspase-1 expressions)

**Figure 3.**
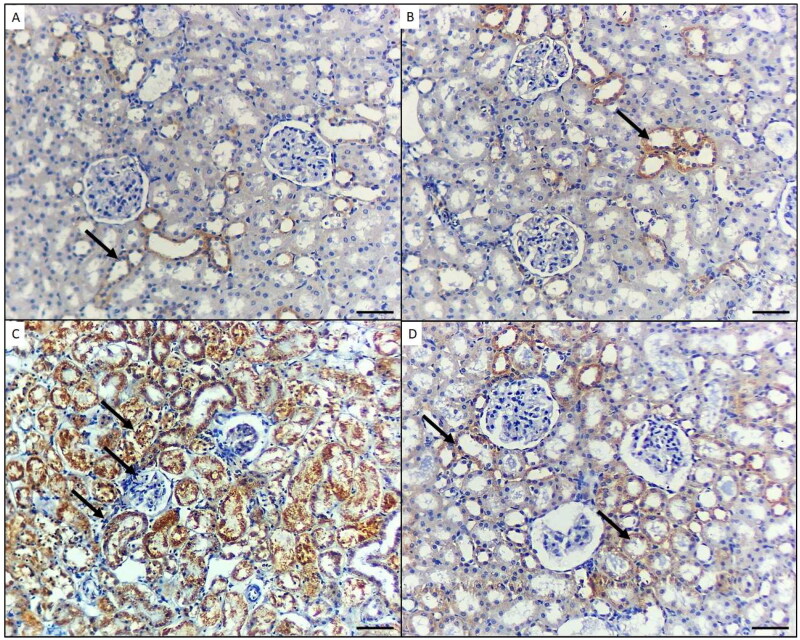
p65 immunohistochemical staining in the kidney of mice in the different groups. A) Control group and B) Mice treated by gallic acid alone showing minimal p65 reactivity in few cells for proximal tubules (arrow). C) Mice treated by NiO-NPs only showing high p65 immunoreactivity in podocytes, proximal and distal tubules cells (arrows). D) Mice treated by NiO-NPs and gallic acid showing minimal p65 immunoreactivity in the proximal tubules cells (arrows). Original magnification 200X, scale bar 50 µm.

Regarding the immunohistochemical expression of NF-κB, the stained kidney tissue from group I and the GA*-*treated mice in group II displayed little to absent NF-κB immunoreactivity (Figure 3A, B). In group III (NiO-NPs-intoxicated), the kidneys’ podocytes and proximal and distal tubular cells exhibited elevated NF-κB immunoreactivity (Figure 3C). In the NiO-NPs- and GA-treated group (group IV), there was mild NF-κB positivity in both the distal and proximal tubular cells (Figure 3D).

**Figure 4.**
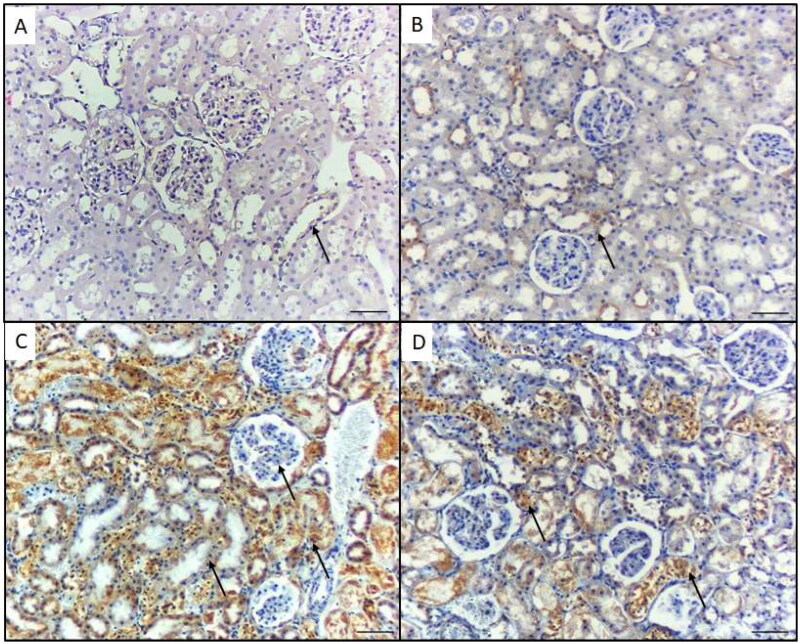
Caspase-1 immunohistochemical staining in the kidney of mice in the different groups. A) Control group and B) Mice treated by gallic acid alone showing minimal Caspase- 1 reactivity in the proximal tubular cells (arrows). C) Mice treated by NiO-NPs only showing high Caspase-1 immunoreactivity in the proximal and distal tubular cells, and podocytes (arrow). D) Mice treated by NiO-NPs and gallic acid showing mild Caspase-1 immunoreactivity in the proximal and distal tubular cells (arrows). Original magnification 200X, scale bar 50 µm.

The immunohistochemical expression of caspase-1 in the stained kidney tissue sections from the controls (group I) and GA-treated mice (group II) showed no immunoreactivity (Figure 4A, B). In the NiO-NPs-intoxicated mice (group III), the proximal and distal tubular cells as well as podocytes in the kidneys displayed highly elevated caspase-1 (Figure 4C). In the NiO-NPs- and GA-treated mice (group IV), both the distal and proximal tubular cells showed mild caspase-1 immunoreactivity (Figure 4D). According to the quantified comparison of immunohistochemical staining of positive area percentage for p65 and caspase-1 in mice different groups, results prove a significant improvement in treated group IV compared with NiO-NPs group III (Table 3).

**Table 3.** The quantified comparison of immunohistochemical staining for P65 and caspase-1 in mice different groups.

Group/ Parameter	Control negative group I	Control negative treated Gallic A. group II	Intoxicated NiO-NPs group III	Intoxicated NiO-NPs group treated Gallic A. group IV	*p* Value
p65 IHC positive area percentage	1.63 ± 1.08	1.18 ± 0.59	47.70 ± 9.74^***¶¶¶^	16.60 ± 5.60^***¶¶¶###^	<0.0001
Caspase-1 IHC positive area percentage	1.71 ± 0.61	1.61 ± 0.52	21.99 ± 7.12^***¶¶¶^	7.68 ± 3.35^**¶¶###^	<0.0001

Data expressed as mean ± SD.

SD: standard deviation; P:probability; Test used: one way ANOVA followed by posthoc Tukey.

*p < 0.05; **p < 0.01; ***p < 0.001 vs. Control negative group I.

¶*p* < 0.05; ¶¶*p* < 0.01; ¶¶¶*p* < 0.001 vs. Control negative treated Gallic A. group II.

#*p* < 0.05; ##*p* < 0.01; ###*p* < 0.001 vs. Intoxicated NiO-NPs group III.

### 4.6. Histopathological results (light microscopic examination of the H&E-stained tissue sections)

**Figure 5.**
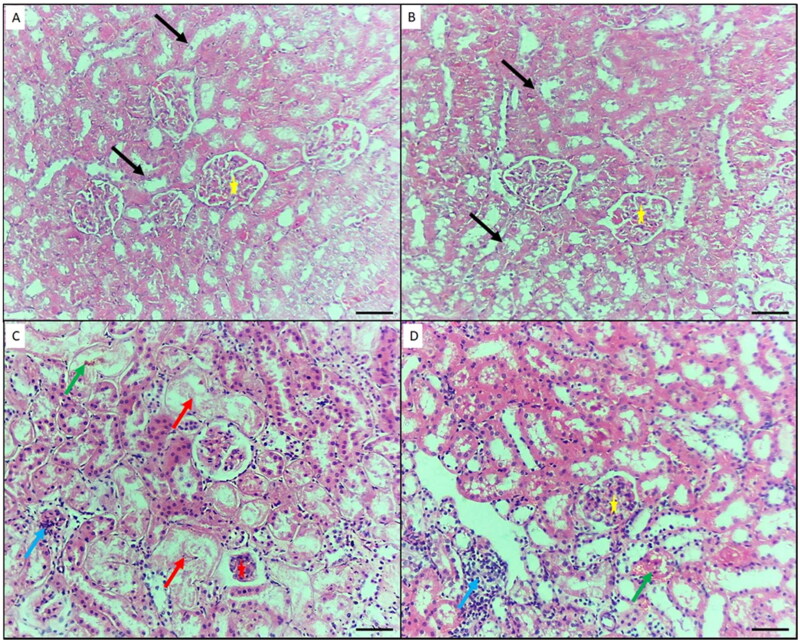
Histopathological changes in the kidney of mice in the different groups. A) Control negative group showing normal features of the kidney; renal convoluted tubules (black arrows), and glomeruli (yellow star). B) Mice treated by gallic acid alone showing normal features of the kidney. C) Mice intoxicated by NiO-NPs only showing extensive renal damage features such as hyaline cast and loss of nuclei in the renal tubules (blue arrow), and shrunken or splitting glomeruli as evident by widened Bowman’s capsule (red star), shrinkage of glomeruli and widening of Bowman’s space (red star), inflammatory cells infiltration (blue arrow), and interstitial hemorrhage (green arrow). D) Mice intoxicated by NiO-NPs and treated gallic acid showing mild renal damage features such as; inflammatory cells infiltration (blue arrow), and interstitial hemorrhage (green arrow). Original magnification 200X, scale bar 50 µm.

Histopathological examination of the kidney tissue samples from the control and GA-treated groups showed normal renal architecture with normal tubules and glomeruli (Figures 5A, B). These findings imply that the experiment was carried out properly and that the mice were in good health. The kidneys of the NiO-NPs-intoxicated mice (group III) revealed a wide range of necrotic characteristics, including a hyaline cast and loss of nuclei in the proximal tubules, cellular debris and denuded epithelium causing intratubular blockage, and a significant infiltration of inflammatory cells (Figure 5C). Concomitant administration of GA and NiO-NPs (group IV) produced mild necrotic characteristics as a result of the denuded epithelium and cellular debris, including intratubular blockage and mild inflammatory cell infiltration (Figure 5D). Moreover, the degree of severity of histopathological changes in kidney sections quantified demonstrate significant reduction in endothelial lesion, glomerular lesion, tubular lesion and interstitial lesion score in GA-treated group IV compared to intoxicated NiO-NPs group III (Table 4).

**Table 4.** The quantified comparison of histopathological parameters study in mice different groups.

Group/ Parameter	Control negative group I	Control negative treated Gallic A. group II	Intoxicated NiO-NPs group III	Intoxicated NiO-NPs group treated Gallic A. group IV	*p* Value
Endothelial lesion score	0.00 ± 0.00	0.00 ± 0.00	2.70 ± 0.48^***¶¶¶^	0.90 ± 0.74^#^	<0.0001
Glomerular lesion score	0.00 ± 0.00	0.00 ± 0.00	2.80 ± 0.42^***¶¶¶^	0.80 ± 0.63^#^	<0.0001
Tubular lesion score	0.00 ± 0.00	0.00 ± 0.00	3.60 ± 0.52^***¶¶¶^	1.10 ± 0.88^#^	<0.0001
Interstitial lesion score	0.00 ± 0.00	0.00 ± 0.00	3.50 ± 0.53^***¶¶¶^	1.10 ± 0.99^#^	<0.0001

Data expressed as mean ± SD.

SD: standard deviation; P: probability; Test used: One way ANOVA followed by post-hoc Tukey.

*p < 0.05; **p < 0.01; ***p < 0.001 vs. Control negative group I.

¶*p* < 0.05; ¶¶*p* < 0.01; ¶¶¶*p* < 0.001 vs. Control negative treated Gallic A. group II.

#*p* < 0.05; ##*p* < 0.01; ###*p* < 0.001 vs. Intoxicated NiO-NPs group III.

## Discussion

Preclinical toxicological investigations have demonstrated that nephrotoxicity presents a significant danger due to the direct destruction of cells and tissues, obstruction of renal excretion, hemodynamic alterations, and inflammation. Important kidney functions include plasma infiltration and the preservation of metabolic homeostasis. Pollutant-induced nephrotoxicity can alter the physiology and structure of the kidney and obstruct renal excretory functions [40].

In this study, we used GA, a potent antioxidant, to investigate its potential nephroprotective benefits against NiO-NPs-induced nephrotoxicity. In our research, NiO-NPs were administered at a dosage of 20 mg/kg body weight. Kidney biomarkers, specifically blood urea nitrogen (BUN) and creatinine levels, were employed as significant diagnostic markers to determine pathophysiological alterations due to the deleterious effects of exposure to toxic elements. The kidney function test revealed a significant increase in the levels of these biomarkers (BUN and creatinine) in group III compared to group I. These changes in kidney biomarkers led to various histopathological alterations. Our findings are consistent with those of Iqbal et al. [41], who reported significant changes in renal biomarkers at high doses in rats with induced kidney toxicities.

Treatment with GA in our study reduced the serum urea and creatinine levels to 21.49 and 0.799 mg/dl, respectively. These results are consistent with the findings of Ahmadvand et al. [26], who demonstrated a significant decrease in creatinine and urea levels and a reduced proteinuria excretion rate in the gentamycin and GA group compared to the gentamycin group.

Our study also highlighted the effects of intraperitoneal injection of NiO-NPs on oxidative stress and lipid peroxidation (LPO) by estimating the tissue levels of superoxide dismutase (SOD), glutathione S-transferase (GST), glutathione peroxidase (GSH-Px) and malondialdehyde (MDA) in mouse kidneys. We observed MDA-induced increases in the group intoxicated with NiO-NPs compared to group I. MDA, the end product of the LPO process [42], is frequently used as a reliable biomarker for assessing oxidative stress caused by various toxicants. Oxidative stress increases the level of ROS activity, which contributes to toxicity caused by NiO-NPs [43,44]. Conversely, the mice that received NiO-NPs showed significant decreases in SOD, GST, and GSH-Px levels, indicating diminished antioxidant capability compared to the GA-treated group IV. According to Yu et al. and Abdulqadir et al. [45,46], the two main mechanisms of toxicity in organs exposed to various toxicants are LPO and decreased antioxidant capability.

Our results align with those of Tammam et al. [47], who found that exposure to NiO-NPs caused a significant increase in MDA content and a significant decrease in catalase (CAT) enzyme activity in the liver and kidney tissues of the NiO-NPs-treated group compared to the control group.

Furthermore, our findings are consistent with those of Razavipour et al. [48], who reported a significant increase in enzyme activity level (*p* = 0.002) in a group that received 25 ppm of NiO-NPs. They also observed a significant decrease in GSH-Px levels in the NiO-NPs-treated group compared to the controls (*p* = 0.012).

Additionally, the results of our study are in line with those of Eslamifar et al. [49]. They concluded that GA therapy mitigated the cisplatin-mediated reduction in GSH in rats (cis group vs. cis-gal group, *p* < 0.01). Compared to the controls, the rats treated with cisplatin exhibited significantly lower antioxidant enzyme activity levels (SOD, *p* < 0.0001 and GSH-Px, *p* < 0.001). These levels significantly increased in the rats treated with GA plus cisplatin compared to those in the cis group (SOD, *p* < 0.01 and GSH-Px, *p* < 0.01).

Our findings demonstrated that the administration of NiO-NPs to group III increased the level of 8-hydroxydeoxyguanosine (8-OH-dG) in the kidney homogenate. This observation is consistent with the findings of Abudayyak et al. [50], who reported that cells incorporated Ni-NPs, which caused dose-dependent DNA damage as assessed by the comet assay and oxidative damage indicated by rising MDA, 8-OH-dG, and other oxidative damage markers.

Mice treated with GA exhibited a decrease in renal 8-OH-dG, a biomarker for DNA damage, compared to those intoxicated with NiO-NPs in group III. According to Kim et al. [51], GA may, therefore, protect against DNA damage by reducing oxidative stress.

Cellular responses to infection, stress, or injury are characterized by inflammation. NiO-NPs can stimulate NF-κB expression through the direct activation of intracellular signal transduction cascades that promote inflammation. Nickel induces apoptosis and activates the NLRP3 inflammasome *via* a mitochondrial ROS-mediated route [52]. In our study, the NiO-NPs-intoxicated group showed significantly higher NF-κB p65 expression levels compared to group I (*p* < 0.001). This finding aligns with the report by Goebeler et al. [53], which states that NiCl2 triggers the transcription levels of proinflammatory cytokines through NF-κB, MAPK, and IRF3 signaling pathways. However, the mice that received GA as a protective antioxidant exhibited only mild NF-κB p65 expression in our experiment. This result is in agreement with Huang et al. [54], who found that GA treatment suppressed p65 phosphorylation and increased p65 deacetylation levels. Consequently, GA modulates the phosphorylation and acetylation of p65 in NF-κB signaling pathways, thereby reducing the production of ADAMTS-4 in nucleus pulposus cells.

Additionally, we measured the expression of NF-κB in the kidney to further investigate underlying processes of GA’s ameliorative effects on NiO-NPs-induced oxidative stress and inflammation. NF-κB is commonly recognized as playing a crucial role in the initiation of inflammatory cytokine transcription, which leads to inflammation and oxidative stress, and is implicated in the pathogenesis of numerous inflammatory illnesses, particularly those affecting the kidney [55].

In the current study, group III, intoxicated with NiO-NPs, showed an increased NF-κB level. These results are consistent with those of Magaye et al. [56], who demonstrated metallic Ni-NPs caused higher activation of AP-1 and NF-κB.

Our findings indicated that GA treatment inhibited the kidney’s production of inflammatory cytokines, such as NF-κB. The potential cause of this effect could be the anti-inflammatory properties of GA within renal tissue. Our results corroborate those of Singla et al. [57], who reported that phosphorylation of p65-NF-κB was found to be reduced (*p* = 0.015) in the GA treated mice group.

In the current study, group III demonstrated a significant increase in protein carbonyl (PC) levels compared to control group I. These results are consistent with those of Poornavaishnavi et al. [58], who found that cells exposed to Ni-NPs showed a significant escalation in LPO and PC.

Furthermore, the NiO-NPs-intoxicated mice in our study exhibited high NF-κB p65 immunoreactivity in the proximal and distal tubular cells and podocytes. This finding aligns with the results of Guo et al. [59], who reported that protein expression levels of NF-κB p65 were significantly higher (*p* < 0.01) in the NiO-NPs-intoxicated group than in group I, based on an immunohistochemical demonstration. However, in group IV treated with GA as a protective antioxidant, our results showed a mild immunohistochemical expression of NF-κB p65. This agrees with the findings of Krajka-Kuzniak and Baer-Dubowska [60], who reported that GA administration reduces the nuclear translocation of NF-κB p65.

Our results also indicated increased caspase-1 expression levels in the immunohistochemical examination of kidney tissues in the NiO-NPs-intoxicated group. This observation is in agreement with Lamkanfi and Dixit’s findings [61], which suggest that NiO-NPs activate caspase-1 expression in response to cellular stresses. The present study also shows that the expression level of caspase-1 was downregulated in the GA-treated group, consistent with the findings of Lin et al. [62], that GA treatment dose-dependently suppressed the release of caspase-1.

Lastly, our histopathological results revealed extensive necrotic features in NiO-NPs intoxicated group, such as the production of hyaline casts, the loss of nuclei in the proximal tubules, severe inflammatory cell infiltration, and intratubular blockage brought on by denuded epithelium and cellular debris. These results are in line with the findings of Xu et al. [63] who observed degenerated kidney tubular cells and apoptotic cells with irregular, shrunken shaped, darkly stained cytoplasm, and condensed nuclei in kidney tissues exposed to NiO-NPs compared to healthy cells.

Moreover, our results confirm the observations reported by Tammam et al. [47]. In their review, they noted that kidney sections from rats exposed to NiO-NPs exhibited abnormal cellular architecture, including large areas of bleeding cast formation, glomerular and tubular congestion, degeneration of the tubular epithelium, desquamation, and brush border loss. The degenerated cells in their study showed irregular geometries, dark-colored cytoplasm, and condensed nuclei compared to healthy cells.

The concomitant administration of GA and NiO-NPs (group IV) resulted in milder necrotic features, such as mild inflammatory cell infiltration and intratubular blockage caused by cellular debris and denuded epithelium. This finding is consistent with that of Obafemi et al. [64], who reported that groups given both aluminum chloride and GA showed normal glomerular architecture, normal proximal and distal convoluted tubules, and a slightly dilated urinary space.

## Conclusion

Our findings strongly suggest that GA improves nephrotoxic parameters, induces kidney tissue regeneration, increases antioxidant activity levels, and regulates protein overexpression in kidneys affected by severe nickel oxide nanoparticles toxicity. These data suggest that GA is a viable and beneficial protective agent in case of kidney injury. Future investigations should aim to further delineate the mechanisms of the GÀs protective effect on kidney injury.

## Data Availability

The data that support the findings of this study are available on request from the corresponding author, El-Refaei MF. The data are not publicly available for the time being and will be available on demand.
